# Supplementary material for: Informed choice of modern Contraceptive Methods and determinant factors among reproductive age women in Eastern Africa countries: A multilevel analysis of demographic and health survey
Source: PLoS One. 2025 May 19;20(5):e0323797. doi: 10.1371/journal.pone.0323797 (PMC12088018; doi:10.1371/journal.pone.0323797)
Supplement: Attachment — Submitted filename: PONE-D-24-06637.pdf [file pone.0323797.s002.pdf]

PLOS ONE

Informed choice of modern Contraceptive Methods and determinant factors among reproductive age women in Eastern Africa countries: A Multilevel Analysis of Demographic and Health Survey.

--Manuscript Draft--

**Manuscript Number:**	PONE-D-24-06637
**Article Type:**	Research Article
**Full Title:**	Informed choice of modern Contraceptive Methods and determinant factors among reproductive age women in Eastern Africa countries: A Multilevel Analysis of Demographic and Health Survey.
**Short Title:**	Informed choice of modern Contraceptive Methods and determinant factors among women in Eastern Africa.
**Corresponding Author:**	gebreeyesus abera zeleke, MSc<br>University of Gondar College of Medicine and Health Sciences<br>Gondar, ETHIOPIA
**Keywords:**	informed choice; Contraceptive Methods; East Africa
**Abstract:**	Background<br><br>Many contraceptive users discontinue their contraceptive methods or fail to use the service; consequently, unwanted pregnancy occurs and this has been public health problem in Sub-Saharan Africa countries especially eastern Africa countries. Informed choice of modern contraceptive method is an important indicator of family planning quality services. Evidence shows that informed choice of contraceptive methods lowers the potential risk of family planning discontinuation rate, misunderstanding of contraceptives method and unintended pregnancies finally lead to induced abortions. Therefore, the aim of this study was to ascertain the magnitude of informed choice of modern contraceptive methods user and its determinant factors among reproductive age women who are currently using selected modern in Easter African countries.<br><br>Methods<br><br>Data from Data from the recent DHS which was covered eight Eastern Africa countries from 2012 to 2020 was used for secondary data analysis. The total weighted sample was 6154 reproductive age women who used were modern contraceptive method. Stata version 14 was used to analysis secondary data. Determinants of informed choice were determined by using multilevel mixed-effects logistic regression model. Significant factors related with informed choice in multilevel mixed effect logistic regression model were decided when the p value of <0.05. The adjusted odds ratio (AOR) and confidence interval (CI) were used to interpret the outcome.<br><br>Result<br><br>In East Africa, the magnitude of informed choice found to be 20.70. determinant factors such maternal age (20 to 35) years (AOR=2.02 CI: 1.39, 2.93), (36-49) years (AOR =2.44, 95% CI: 1.66, 3.61), attending secondary & higher education (AOR =1.37 95% CI 1.02, 1.84), Media exposure (AOR = 1.25 95% CI 1.05, 1.49), visit health facility within 12 months were (AOR =1.31 95% CI: 1.11, 1.54) were significantly associated with an informed choice among reproductive age women modern contraceptive method user.<br><br>Conclusions and recommendation<br><br>This study finalized that an informed choice over eight Eastern Africa countries among reproductive age women who used modern contraceptive methods were low. Maternal age, attending secondary & higher education, Media exposure, visit health facility within 12 months were significantly associated with informed choice. Therefore, The Government, ministry of health and other stake holders in eastern Africa countries shall designing policies and strategies targeting informed choice modern contraceptive

	methods and above significant factors among reproductive age women from 15-49 years in Eastern Africa
Order of Authors:	gebreeyesus abera zeleke, MSc
	Gebreeyesus Atena Zeleke, MSc
	Gebreeyesus Belay Zeleke
	Gebreeyesus Zeleke, MSc
	Gebreeyesus Tarik Zeleke, MSc
	Gebreeyesus Zemene Zeleke, PhD
	Gebreeyesus Ferede Zeleke, MSc
Additional Information:	
Question	Response
**Financial Disclosure**<br><br>Enter a financial disclosure statement that describes the sources of funding for the work included in this submission. Review the submission guidelines for detailed requirements. View published research articles from *PLOS ONE* for specific examples.<br><br>This statement is required for submission and **will appear in the published article** if the submission is accepted. Please make sure it is accurate.<br><br>**Funded studies**<br>Enter a statement with the following details:<br>• Initials of the authors who received each award<br>• Grant numbers awarded to each author<br>• The full name of each funder<br>• URL of each funder website<br>• Did the sponsors or funders play any role in the study design, data collection and analysis, decision to publish, or preparation of the manuscript?<br><br>Did you receive funding for this work?	The author(s) received no specific funding for this work.
**Competing Interests**<br><br>Use the instructions below to enter a competing interest statement for this submission. On behalf of all authors, disclose any competing interests that could be perceived to bias this	Authors Declared that, there is no competing interest

work—acknowledging all financial support and any other relevant financial or non-financial competing interests.<br><br>This statement is required for submission and **will appear in the published article** if the submission is accepted. Please make sure it is accurate and that any funding sources listed in your Funding Information later in the submission form are also declared in your Financial Disclosure statement.<br><br>View published research articles from *PLOS ONE* for specific examples.<br><br>**NO authors have competing interests**<br><br>Enter: *The authors have declared that no competing interests exist.*<br><br>**Authors with competing interests**<br><br>Enter competing interest details beginning with this statement:<br><br>*I have read the journal's policy and the authors of this manuscript have the following competing interests: [insert competing interests here]*<br><br>* typeset	
**Ethics Statement**<br><br>Enter an ethics statement for this submission. This statement is required if the study involved:<br><br>• Human participants<br>• Human specimens or tissue<br>• Vertebrate animals or cephalopods<br>• Vertebrate embryos or tissues<br>• Field research<br><br>Write "N/A" if the submission does not require an ethics statement.	This study was based on analysis of existing survey datasets in the public domain (EDHS) that are freely available online with all the identifier information anonymized, no ethical approval was required.

Powered by Editorial Manager® and ProduXion Manager® from Aries Systems Corporation

General guidance is provided below. Consult the submission guidelines for detailed instructions. **Make sure that all information entered here is included in the Methods section of the manuscript.**<br><br>**Format for specific study types**<br><br>**Human Subject Research (involving human participants and/or tissue)**<br>• Give the name of the institutional review board or ethics committee that approved the study<br>• Include the approval number and/or a statement indicating approval of this research<br>• Indicate the form of consent obtained (written/oral) or the reason that consent was not obtained (e.g. the data were analyzed anonymously)<br><br>**Animal Research (involving vertebrate animals, embryos or tissues)**<br>• Provide the name of the Institutional Animal Care and Use Committee (IACUC) or other relevant ethics board that reviewed the study protocol, and indicate whether they approved this research or granted a formal waiver of ethical approval<br>• Include an approval number if one was obtained<br>• If the study involved *non-human primates*, add *additional details* about animal welfare and steps taken to ameliorate suffering<br>• If anesthesia, euthanasia, or any kind of animal sacrifice is part of the study, include briefly which substances and/or methods were applied<br><br>**Field Research**<br><br>Include the following details if this study involves the collection of plant, animal, or other materials from a natural setting:<br>• Field permit number<br>• Name of the institution or relevant body that granted permission	
**Data Availability**<br><br>Authors are required to make all data underlying the findings described fully	No - some restrictions will apply

available, without restriction, and from the time of publication. PLOS allows rare exceptions to address legal and ethical concerns. See the PLOS Data Policy and FAQ for detailed information.<br><br>A Data Availability Statement describing where the data can be found is required at submission. Your answers to this question constitute the Data Availability Statement and **will be published in the article**, if accepted.<br><br>**Important:** Stating 'data available on request from the author' is not sufficient. If your data are only available upon request, select 'No' for the first question and explain your exceptional situation in the text box.<br><br>Do the authors confirm that all data underlying the findings described in their manuscript are fully available without restriction?	
**Describe where the data may be found in full sentences. If you are copying our sample text, replace any instances of XXX with the appropriate details.**<br><br>• If the data are **held or will be held in a public repository**, include URLs, accession numbers or DOIs. If this information will only be available after acceptance, indicate this by ticking the box below. For example: *All XXX files are available from the XXX database (accession number(s) XXX, XXX.*).<br>• If the data are all contained **within the manuscript and/or Supporting Information files**, enter the following: *All relevant data are within the manuscript and its Supporting Information files.*<br>• If neither of these applies but you are able to provide **details of access elsewhere**, with or without limitations, please do so. For example:<br><br>*Data cannot be shared publicly because of [XXX]. Data are available from the XXX Institutional Data Access / Ethics*	no

Committee (contact via XXX) for researchers who meet the criteria for access to confidential data.<br><br>*The data underlying the results presented in the study are available from (include the name of the third party and contact information or URL).*<br>• This text is appropriate if the data are owned by a third party and authors do not have permission to share the data.<br><br>* typeset	
Additional data availability information:	

Powered by Editorial Manager® and ProduXion Manager® from Aries Systems Corporation

Manuscript

Click here to access/download;Manuscript;INFORMED CHOICE Q1.docx

Informed choice of modern Contraceptive Methods and determinant factors among reproductive age women in Eastern Africa countries: A Multilevel Analysis of Demographic and Health Survey.

Gebreeyesus Abera Zeleke[1*], Birtukan Atena Negash[1], Kassaw Belay Shiferaw[2], Berhan Tekeba[3], Tadesse Tarik Tamir[3], Workie Zemene Worku[4], Alebachew Ferede Zegeye[5].

[1] Department of Surgical Nursing, School of Nursing, College of Medicine and Health Science, University of Gondar, Gondar, Ethiopia.

[2] Department of physiotherapy, school of medicine College of Medicine and Health Sciences University of Gondar, Gondar, Ethiopia.

[3] Department of Pediatric and Child Health Nursing, School of Nursing, College of Medicine and Health Sciences, University of Gondar, Gondar Ethiopia.

[4] Department of Medical Nursing, School of Nursing, College of Medicine and Health Sciences University of Gondar, Gondar, Ethiopia.

[5] Department of community nursing, School of Nursing, College of Medicine and Health Science, University of Gondar, Ethiopia.

Correspondence Author*

Gebreeyesus Abera Zeleke

Department of Surgical Nursing, School of Nursing, College of Medicine and Health Science, University of Gondar, Gondar, Ethiopia.

Email address: gebreeyesusabe143@gmail.com

Co-authors

Birtukan Atena Negash

Email Address: birtukanatena@gmail.com

Kassaw Belay Shiferaw

Email address: uogbelay@gmail.com

Berhan Tekeba

Email address: berishboss7@gmail.com

Tadesse Tarik Tamir

Email address: Tadestar140@gmail.com

Workie Zemene Worku

Email Address: workiezemene@gmail.com

Alebachew Ferede Zegeye

Email address: alexferede24@gmail.com

Abstract

Background: Unwanted pregnancies arise from the discontinuation of many contraceptive techniques or the failure to use current contraceptive services; this has been a public health concern in Sub-Saharan Africa, particularly in the eastern African countries. Informed choice of modern contraceptive method is an important indicator of family planning quality services. Evidence shows that informed choice of contraceptive methods lowers the potential risk of family planning discontinuation rate, misunderstanding of contraceptives method and unintended pregnancies finally lead to induced abortions. Therefore, the aim of this study was to ascertain the magnitude of informed choice of modern contraceptive methods user and its determinant factors among reproductive age women who are currently using selected modern in Easter African countries.

Methods: Data from Data from the recent DHS which was covered eight Eastern Africa countries from 2012 to 2020 was used for secondary data analysis. The total weighted sample was 6154 reproductive age women who used were modern contraceptive method. Stata version 14 was used to analysis secondary data. Determinants of informed choice were determined by using multilevel mixed-effects logistic regression model. Significant factors related with informed choice in multilevel mixed effect logistic regression model were decided when the p value of <0.05. The adjusted odds ratio (AOR) and confidence interval (CI) were used to interpret the outcome.

Result: In East Africa, the magnitude of informed choice found to be 20.70. determinant factors such maternal age (20 to 35) years (AOR=2.02 CI: 1.39, 2.93), (36-49) years (AOR =2.44, 95% CI: 1.66, 3.61), attending secondary & higher education (AOR =1.37 95% CI 1.02, 1.84), Media exposure (AOR = 1.25 95% CI 1.05, 1.49), visit health facility within 12 months were (AOR =1.31 95% CI: 1.11, 1.54) were significantly associated with an informed choice among reproductive age women modern contraceptive method user.

Conclusions and recommendation: This study concluded that the proportion of reproductive age women using modern contraceptive techniques made an informed choice in eight nations in Eastern Africa. The following factors were strongly linked to informed choice: maternal age, attending secondary and higher education, media exposure, and visiting a health facility within a year. Therefore, policies and initiatives targeting informed choice modern contraceptive methods and above critical determinants among reproductive age women in Eastern Africa (ages 15 to 49) will be designed by the government, ministry of health, and other relevant parties.
Keywords: East Africa, contraceptive methods, informed decision

.

Background

Family planning access has remained a fundamental component of services for sexual and reproductive health, according to in Cairo declaration, International Conference on population Development(1). The term "family planning" refers to all the services, including contraception, that assist individuals in preventing unintended pregnancies, deciding whether to have children, and deciding when to do so(2). People fundamentally have the right to obtain contraceptive information and services, and high-quality contraceptive information and services support people's autonomy to decide how many children to have and how spaced apart to have them, as well as offering a number of potential benefits, such as better mother and child health(3). The freedom of a person or couple to make a freely chosen decision based on possibilities that they have a thorough understanding of, maybe through comprehension knowledge, is known as informed choice(4). Unwanted pregnancies result from many contraceptive users quitting their methods or failing to utilize them, which is a public health concern in Sub-Saharan African nations, particularly those in Eastern Africa. (5). A key indicator of high-quality family planning services is the ability to make an informed decision about a contemporary contraceptive method. (6, 7). Evidence shows that informed choice of contraceptive methods lowers the potential risk of family planning discontinuation rate, dissatisfaction of contraceptives methods, unintended pregnancies which finally lead to induced abortions(8, 9). Informed choice has been crucial in assisting people in choosing a method that best suits their requirements and expectations in order to encourage the efficient use of contraceptives **(10).** There is very little evidence that informed choosing of contemporary contraceptive methods is commonplace in various parts of the world. A study done in India 15.6%(11), Bangladesh 22%(12), Kenya 55.5%(13), Ethiopian (EDHS 2016) (36.2%)(14), in sub-Saharan Africa 49.47%(5).previous research on the factors that influence the informed choice of contraceptive methods has shown that the following factors significantly influence the informed choice of contraceptive methods: husband's occupation, residence, types of contraceptive use, wealth index, media exposure, maternal education, source of contraceptive method, and visits to health facilities within the last year.(11, 14-16) Diverse approaches have been used worldwide to boost the use of contraceptives; nonetheless, the incidence of these techniques in Sub-Saharan Africa (SSA) is still unacceptably low, and the

false perception of their side effects is a serious public health issue. (17, 18). As far as we are aware, there is very little information on the informed choice of contraceptive methods in the eastern African region, despite the fact that studies have been done on the utilization, unmet need, and discontinuation of contraceptive methods in SSA nations.

Therefore, the aim of this study was to ascertain the magnitude of informed choice of modern contraceptive methods user and its determinants among women aged 15-49 years who are currently using selected modern such as (Pill, injectable, IUD, Norplant/ implant, female sterilization) in Easter African countries

Methods

Patient and public involvement statement

The data used in this study were secondary data from the Demographic and Health Surveys (DHS).

Study design, study area, and period

According to the most current demographic health study Multilevel mixed effect analysis was performed using data from the eight East African nations where research was done between 2012 and 2020. Every five years, the DHS collects community-based cross-sectional research data in order to generate updated health and health-related indicators.

Data source, study population and sampling technique

The secondary data analysis was conducted using the DHS statistics for East African nations from 2012 to 2020. We made use of DHS surveys from eight nations in East Africa: Burundi, Comoros, Ethiopia, Malawi, Rwanda, Uganda, Zambia, and Zimbabwe. The data were appended to filter out the magnitude and determinant factors of among reproductive age women who were informed choice among modern contraceptive usage in East African nations. The survey for every country contains different datasets. DHS deploys a stratified two-stage cluster design that includes enumeration areas as the first stage and generates a sample of households from each enumeration area as the second stage. The variable modern contraceptive (v312) from maternal recode (IR) data set was recoded to drop other than pill IUD, injectable, female sterilization, implant/Norplant contraceptive methods. A binary logistic regression model was used to identify determinants a factor to informed choice of modern contraceptive users. Determinants of informed choice were reported in terms of an AOR with a significance level of (95%). In the univariate analysis, at 95%

confidence intervals with a p-value of < 0.25 was considered a candidate for the multivariable analysis of data. All variables with p values <0.05 were considered statistically significant. A total weighted sample of 6154 women was included in the study (**Table 1**)

Table 1: The weighted Sample size for magnitude and determinant factors of among reproductive age 15-49 years women with modern contraceptive user in Eastern Africa countries.

**Country**	**years of survey**	**weighted sample (n)**	**weighted sample (%)**
Burundi	2016/2017	442	7.2
Ethiopia	2016	1454	23.7
Comoros	2012	99	1.61
Malawi	2015/2016	948	15.45
Rwanda	2019/2020	905	15
Uganda	2016	909	15
Zambia	2018	490	7.98
Zimbabwe	2015	887	14.46
**Total**		**6154**	**100%**

Study variable

Outcome variables: An informed choice of contemporary methods of contraception was the study's outcome. The six contemporary methods of contraception—female sterilization, implant/Norplant, injectable contraceptive, IUD, pill, and emergency contraceptive—were the subjects of a study on informed choice making among reproductive individuals aged 15 to 49. The DHS questionnaire was utilized to develop it, with responses obtained from the participants. Four factors were computed to define how the outcome variables should be measured **(table 2).**

Table 2: Determination and computation Outcome Variables Used in the DHS datasets.

Variable	Explanation	Measurement
Informed about side effect	**when Started the current method,**<br>1.were you told about side effects?<br>2.Were you ever told by a health or family-planning worker about side effects.	Yes=1 (informed)<br>No = 0 (not informed)
Informed other available method	**When you started the current method,**<br>3. were you told about other methods of family planning that you could use?<br>4. Were you ever told by a health or family-planning worker about other methods of family planning that you could use?	Yes=1 (informed)<br>No=0 (Not informed)
Informed choice for modern contraception	Percentage of women who were informed about both side effects and other available methods	0 =No<br>1=Yes

Independent Variable: Hence DHS data are hierarchical, independent factors come from two sources that was at individual and community levels were considered for this analysis. The individual-level independent variables were reproductive women's age (15-19, 20-35, 36-49), Maternal educational status (No formal education, Primary education , Secondary and Higher education), Maternal occupation (House wife, employee, Others,), Religion (Cristian, Muslim, Others), Wealth index (Poor, Middle, Distance to health facility (Big problem, Not a big problem), media exposure (yes, no), intermate utilization (yes, no), Residency (urban, rural), Husband educational status (No formal education, Primary education, secondary and higher education) Occupational status (No working, Employed, house wife, Other), Husband occupational status (No

working, Employed, Other), Contraceptive Decision (women, husband/partner, Joint decision), Currently Contraceptive (No, Yes) Visiting health facility in 12 month (No, Yes)

The community-level variables: Community poverty level (Low, High), Community illiteracy level (Low, High), Community media exposure, (Low, High), Community distance from health facility (Low, High), Place of residency (Urban, rural) in East African countries.

Data processing and statically analysis

Stata version 14 statistical software was used to record, clean, and analyze the data that were taken from DHS data sets. The variables in the DHS data are arranged into clusters, with the similarity between variables inside a cluster being greater than that of variables outside of it. In order to do a conventional logistic regression analysis, the presumptions of independent data and uniform variance among clusters were violated. This suggests that taking in to account between-cluster effects requires the use of a complex model. In light of this, multilevel mixed-effects logistic regression was employed to identify the variables linked to knowledgeable selection of contemporary contraceptives.

Multilevel mixed effect logistic regression follows four models: the null model (only outcome variable), mode I (individual level variables), model II (community level variables), and model III (both individual and community level variables). The null model without independent variables was used to check the variability of informed choice of modern contraceptive method across the cluster. The relationship of individual-level variables with outcome variable (Model I) and the association of community-level variables with outcome variable (Model II) were determined. In the final model, the association of both individual and community-level variables was fitted with the outcome variable.

Random effects

The intra-class correlation coefficient (ICC), proportional change in variance, and median odds ratio (MOR) were used to assess random effects or measures of variation of the outcome variables (PCV) To quantify the variation between clusters, the intra-cluster correlation coefficient (ICC) and proportionate change in variance (PCV) were calculated. When considering each cluster as a random variable, the ICC shows that the informed choice variance between clusters was computed as; $ICC = \frac{VC}{VC+3.29} \times 100\%$ (19). The MOR is the median value of the odds ratio between the area of the highest risk and the area of the lowest risk for informed

choice of modern contraceptive when two clusters are randomly selected, using clusters as a random variable; MOR= $e^{0.95\sqrt{VC}}$. (19). In addition to this, the PCV reveals the variation in the magnitude of informed choice of modern contraceptive usage explained by factors and computed as; $PCV = \frac{Vnull-VC}{Vnull} \times 00\%$; (19)where Vnull = variance of the null model and VC = cluster level variance (20-22). The likelihood of making an informed decision and independent variables at the individual and community levels were estimated using the fixed effects. Adjusted odds ratio (AOR) and 95% confidence intervals with a p-value of less than 0.05 were used to evaluate and present the data. Deviance = -2 (log likelihood ratio) was used to compare the models due to the nested nature of the model, and the model with the lowest deviance was chosen as the best-fitted model. By evaluating the variance inflation factors (VIF), the multi-collinearity of the variables employed in the models was confirmed, and the results were found to be within an acceptable range of 1–10.

Result

This study included 6154 women of reproductive age who made an informed decision to use a modern type of contraception. Over two thirds (71.18%) of the women in the survey were from rural East Africa, and over half (51.9%) had only completed their primary education. Additionally, over 90% of participants did not use the internet, and over half (65.45) of the women had visited a health facility in the year prior to the survey **(table 3)**

Table 3: sociodemographic and economic characteristics of reproductive women who had informed choice modern contraceptive user in East Africa countries**.**

**Variable**	**frequency(n)**	**percentage (%)**
**Individual and community level variables**		
**Age in year**		
15-19	453	8.08
20-35	3852	68.73
36-49	1299	23.17
**Residency**		
Urban	1,615	28.81
Rural	3989	71.18
**Religion**		
Cristian	3243	57.87
Muslim	709	12.65
other	1652	29.47
**Educational Status**		
No formal education	1041	18.57
Primary education	2909	51.9
Secondary and high	1654	29.51
**Husband educational status**		
No formal education	790	16.38
Primary education	2165	44.9
secondary and higher	1867	38.71
**Occupational status**		
No working	1442	25.73
Employed	2091	37.31
house wife	81	1.44
Other	830	18.81
**Husband occupational status**		
No working	353	6.29

Employed	2517	44.91
Other	2737	48.84
**Wealth index**		
lower	2101	37.49
middle	1060	18.91
higher	2443	43.59
**Media exposure**		
No	2053	36.63
Yes	3551	63.36
**Intermate utilization**		
No	5,009	90.99
Yes	496	9.00
**Contraceptive Decision**		
Female	1009	20.94
Male/partner	343	7.12
Joint decision	3465	71.93
**Currently Contraceptive**		
No	4263	78.42
Yes	1180	21.67
**Visiting health facility 12 month**		
No	1,936	34.54
Yes	3668	65.45
**Distance from health facility**		
Big problem	2113	37.70
Not big problem	3491	62.29
**community poverty level**		
Low	2914	51.99
High	2690	48.00
**Community illiteracy level**		
Low	2332	41.61
High	3227	57.58
**Community media exposure**		
Low	2200	39.25
High	3404	60.74
**Community distance health facility**		
Low	2716	48.46
High	2888	51.53

The magnitude of informed choice among reproductive age women who used modern contraceptive method such as pill, IUD, injectable, implant/Norplant in Eastern African countries found to be 20.70 (95% CI: (19.65, 21.78). (**Fig 1:)**

Random effect (Measures of variation) and model fitness

To determine whether the data supported the choice to evaluate randomness at the community level, a null model was employed. Significant variations in informed choice were found between communities according to the null model's results, which had a variance of.0883961 and a P value of 0.000. While the variance across clusters accounted for 2.61% of the overall cluster variation, the variance within clusters provided 11.56%. The probabilities of making an educated decision varied by a factor of times in the null model between higher and lower risk clusters. According to Model I's intraclass correlation value, the differences between communities are explained by 0.76% of the variation in informed choice. Next, we created Model II using community-level variables and the null model. The ICC value from Model II indicated that 16.61% of the differences in informed choice were due to cluster variability. The likelihood of informed choice varied 1.69 times between low and high informed choice in clusters in the final model (model III), which ascribed both individual and community-level variables to about 79.44% of the variation in the likelihood of informed choice.

Table 4: model comparison and random effect analysis for informed choice of modern contraceptive among reproductive age women in East African countries.

Parameter	Null model	model I	model II	model III
Variance	.0883961	0.053887	0.655594	0.430142
ICC	2.61%	1.61%	16.61%	11.56%
MOR	0.76	.60	2.09	1.69
PCV	Reference	64.03%	86.52%	79.44

Table 5:

Model fitness

LLR	-2855.5044	-2353.2748	-2839.6822	-2349.9148
Deviance	5711.008	4706.5496	5679.3644	4699.8296

ICC: intra-cluster correlation, LLR: loglikelihood ratio, MOR: median odds ratio, PCV: proportional change in variance.

Determinants of individual and community level factors with informed choice of among reproductive age women who used modern Contraceptive in East Africa countries.

Maternal ages of 20–35, 36–49, secondary and higher education, media exposure, and visiting a health facility were found to be significantly associated with informed choice of modern contraceptive among reproductive age women in east African countries, according to the final fitted model of multivariable multilevel logistic regression.

The odds of informed choice were 2.022 times higher among reproductive women aged 20 to 35 years compared to women aged 15to 19 years **(AOR** = 2.02 95% CI: 1.39-2.92). The odds of informed choice were 2.44 times more likely to occur among reproductive women age 15 to 19 years of age **(AOR**=2.44, 95% CI, 1.66-3.61) and the odds of informed choice were 1.37 times higher who had secondary and higher education level compared to women who had no any education **(AOR=**1.37 95% CI 1.02-1.84). A reproductive age woman who had media exposure were 1.25 times more likely to informed choice for modern contraceptive compared to a woman who had not media exposure **(AOR**=1.25 95% CI: (1.05-1.49). and women who were visiting health facility 1.32 times more likely informed choice for modern contraceptive compared to women who were no visiting health facility with in 12 months of study period **(AOR=**1.32 95% CI (1.11-1.54).

Table 6: Multivariable multilevel logistic regression analysis of individual-level and community level variable associated with informed choice among reproductive women who use modern contraceptive in East African countries.

**Individual and community level factors**	**Model I AOR (95% CI)**	**Model II AOR (95% CI)**	**Model III AOR (95% CI)**
**Maternal age**			
15-19	1		**1**
20-35	2.01(1.39, 2.91)		**2.02 (1.39, 2.93) ****
36-49	2.45 (1.66, 3.62)		**2.44 (1.66, 3.61) ****
**Residency**			
Urban		0.79 (0.69, 0.92)	1.13 (0.92-1.40)
Rural		1	1
**Maternal educational level**			
No education	1		1
primary education	1.22 (0.97, 1.54)		1.19 (0.94, 1.5)

secondary and higher	0.20 (1.06, 1.89)		**1.37 (1.02, 1.84) ** **
**Husband education**			
No education	1		1
Primary education	0.89 (0.77, 1.25)		0.98(0.77, 1.25)
Secondary & higher	0.15 (0.83, 1.45)		1.08(0.82, 1.42)
**Marital occupation**			
No working	1		1
employed	1.05 (0.99, 1.25)		1.04(0.87, 1.24)
house wife	0.71 (0.33, 1.50)		0.7(0.33, 1.49)
Other	1.10 (0.89, 1.37)		1.08 (0.86, 1.34)
**Husband occupation**			
no working	1		1
employed	0.78 (0.45, 1.50)		1.14 (0.84, 1.55)
other	0.74 (0.43, 1.78)		1.15 (0.84, 1.57)
**Wealth index**			
lower	1		1
middle	1.04 (0.84, 1.28)		1.06 (0.86, 1.31)
rich	0.93 (0.76, 1.15)		0.95 (0.77, 1.18)
**Media Exposure**			
No	1		1
Yes	1.28 (1.08, 1.53)		**1.25 (1.05, 1.49**) **
**Internet use**			
No	1		1
Yes	1.11(0.85, 1.47)		1.14 (0.84, 1.46)
**Decision maker on Contraceptive**			
women	1		1
Husband/partner	1.26(0.91, 1.72)		1.26 (0.92, 1.73)
Joint destion	1.07 (0.89, 1.29)		1.08 (0.90, 1.30)
**Health facility visiting status**			
No	1		1
Yes	1.32 (1.12, 1.55)		**1.31 (1.11, 1.54) ** **
**Distance from health facility**			
Big problem	1		1
Not big problem	0.90 (0.77, 1.06)		0.90 (0.76, 1.75)
**Community level variable**			
**Community poverty level**			
Low		1	1
High		1.17 (1.00, 1.37)	1.15 (0.97, 1.37)
**Community illiteracy level**			

low	1	1
High	0.78 (0.68, 0.91)	0.87 (0.74, 1.02)
**Community Media Exposure**		
Low	1	1
High	0.88 (0.75, 1.03)	0.88 (0.74, 1.04)
**Community distance health facility**		
Low	1	1
High	0.98 (0.85, 1.14)	1.03 (0.88, 1.21)
**East African countries**		
Low income	1	1
lower middle-income countries	1.11 (0.96, 1.27)	1.03 (0.87, 1.21)

Discussion

Informed choice of modern contraceptive method contributes significant problem among reproductive age women in developing countries mainly in Eastern Africa. The aim of this research was to ascertain the magnitude and determinant factors modern contraceptive informed choice among reproductive age women in Eastern Africa countries. In this study, the magnitude of informed choice among reproductive age women found to be 20.70 (95% CI: (19.65, 21.78). This finding revealed that substantial number of reproductive age women using modern contraception method were uninformed choice about contraceptive methods, side effects and other concerned issue of family planning, consequently, discontinuation of service use(23). This finding was lower than the previous studies conducted in sub-Saharan Africa 49.47%(5) , multilevel study in Ethiopia 36.2%(14), 30% (24) UNFPA study in 24 countries 52%(25) . This discrepancy might be due to the deference in socio-econommic and demographic condition, and variation in health infrastructure and health care service policy and the health service coverage, quality of maternal healthcare services, and economic and health policies of UNFPA the are better compared with those of Eastern Africa countries. On the other hand, this study was inline with study conducted southern Ethiopia , Sidama Region 23.5%(26)

In the final model of multilevel logistic regression analysis variables such as Maternal age, Maternal education, having media exposure, had visiting health facility within 12 months were found to be significantly associated with informed choice of modern contraceptive method user. Given this, the odds of having an informed choice of modern contraceptive among women 20-35 and 36-49 years old were 2.02 times (AOR=2.02 CI: 1.39, 2.93) and 2.44 times (AOR =2.44, 95% CI: 1.66, 3.61) higher as compared to 15-19 years old women respectively. This find was consistent with study done in Ethiopia (14), sub-Saharan countries (5) , India(11). As the women's age increases, the chance of having an information, experience and learning about contraceptive also increases also one study conclude that, healthcare providers marginalize attitudes toward younger women(11, 27). At the same time, women who attend secondary & higher education were 1.37 times (AOR =1.37 95% CI 1.02, 1.84) higher odds of informed choice of modern contraceptive methods user compared to women who had no formal education. This is consistent, with studies done elsewhere (5, 11, 14, 28). The possible reason could be higher educated women more likely to engage with health care provider communication and understand information's they receive

from their health care provider about informed choice of modern contraceptive, in addition, Educated women were better information access from other sources like media (15, 29). This result indicated that the importance of womens education for enhancing informed choice of modern contraceptive methods in East African countries.

Similarly, women who had media exposure were 1.25 times (AOR = 1.25 95% CI 1.05, 1.49) higher odds of informed choice of modern contraceptive methods user as compared to women who had no media exposure. This finding was similar with study done in Ethiopia (EDHS 2016) and sub Saharan Africa (5, 30). The possible justification for thus association was, women who had media exposure could be easily retained information about different types of contraceptive methods and their side effects, which enabled them to make an informed of contraceptive methods(31). In addition to this, women who visit health facility within 12 months were 1.31 times (AOR =1.31 95% CI: 1.11, 1.54) higher odds of informed choice of modern contraceptive methods user as compared to those who were not visiting health facility within 12 months of survey. This finding was consistent with study in Ethiopia (EDHS 2016). The justification of this finding could be, women's who had trained visiting health facility could easily obtain or access more information about different types of contraceptive methods, their preferable choice about contraception(31).

Strength and limitation

Data from national health surveys and demographic surveys were used in this investigation. As with any cross-sectional study, there are limitations. First, the findings on informed choice only include a subset of modern contraceptive methods, such as pills, IUDs, implants, implants/Norplant, injectables like Depo-Provera, and female sterilization. Moreover, recall bias was not avoided and a causal relationship was not determined.

. Conclusion and Recommendation

Many East African women between the ages of 15 and 49 who are fertile were not well informed about the harmful effects of contemporary contraceptives or other options. Merely 20.7% of the women in this survey had sufficient knowledge about the negative effects of contemporary contraceptives and the options that healthcare practitioners may supply. The following factors were found to be significantly associated with an informed choice of contraceptive methods: maternal age, maternal educational status, media exposure, and visiting a health facility within a year. As a result, health care facilities should prioritize empowering women to make an informed choice in order to ensure that the service is continued. In order to assist women in making informed decisions about contraceptive options, providers will also use counseling sessions.

Declaration

Ethical approval and consent to participate

There was no need for ethical approval because this study was based on an analysis of survey datasets that were already in the public domain (EDHS) and readily accessible online with all identifying information anonymized.

Consent for publication

Not applicable

Data and material availability

Demographic and Health Survey were used, and it is publicly available online at (https://www.dhsprogram.com).

Competing interest

Authors Declared that, there is no competing interest.

Funding

Not applicable

Author's contributions

GAZ: involved in designing the study, data extraction, data analysis, interpretation, report and manuscript writing.

BAN: involved in data analysis, interpretation, and manuscript writing.

KBS: involved in data extraction, and manuscript writing.

BT: involved in analysis, and result interpretation.

TTT: involved in designing the study, interpretation, report and manuscript writing

WZW: involved in analysis, and result interpretation

AFZ: involved in designing the study, interpretation analysis and manuscript writing

Acknowledgements

We would like to say the DHS programs for allowing us to use the relevant DHS dataset in this study.

Reference

1. Rimon JG, Tsui AOJGHS, Practice. Regaining momentum in family planning. 2018;6(4):626-8.
2. Starbird E, Norton M, Marcus RJGhs, practice. Investing in family planning: key to achieving the sustainable development goals. 2016;4(2):191-210.
3. Ali M, Tran NTJBS, Health R. Defining counselling in contraceptive information and services: outcomes from an expert think tank. British Medical Journal Publishing Group; 2021.
4. Newman K, Feldman-Jacobs CJW, DC: Population Reference Bureau. Family Planning and Human Rights–What's the connection and why is it important. 2015.
5. Tsega NT, Haile TT, Asratie MH, Belay DG, Endalew M, Aragaw FM, et al. Pooled prevalence and determinants of informed choice of contraceptive methods among reproductive age women in Sub-Saharan Africa: A multilevel analysis. 2022;10:962675.
6. Hancock NL, Stuart GS, Tang JH, Chibwesha CJ, Stringer JS, Chi BHJC, et al. Renewing focus on family planning service quality globally. 2016;1:1-6.
7. Polis C, Bradley SE, Bankole A, Onda T, Croft TN, Singh S. Contraceptive failure rates in the developing world: an analysis of demographic and health survey data in 43 countries. 2016.
8. Chakraborty NM, Chang K, Bellows B, Grépin KA, Hameed W, Kalamar A, et al. Association between the quality of contraceptive counseling and method continuation: findings from a prospective cohort study in social franchise clinics in Pakistan and Uganda. 2019;7(1):87-102.
9. Kisuza RK, Kicaber S, Bary DA, Bongomin F, Garimoi COJm. Factors associated with contraceptive failure in Uganda: Analysis of the 2016 Uganda Demographic and Health Survey. 2022:2022.02. 09.22270716.
10. Dehlendorf C, Krajewski C, Borrero SJCo, gynecology. Contraceptive counseling: best practices to ensure quality communication and enable effective contraceptive use. 2014;57(4):659.
11. Jain AKJJobs. Information about methods received by contraceptive users in India. 2017;49(6):798-810.
12. Hossain S, Sripad P, Zieman B, Roy S, Kennedy S, Hossain I, et al. Measuring quality of care at the community level using the contraceptive method information index plus and client reported experience metrics in Bangladesh. 2021;11.
13. Feeser K, Chakraborty NM, Calhoun L, Speizer ISJGor. Measures of family planning service quality associated with contraceptive discontinuation: an analysis of Measurement, Learning & Evaluation (MLE) project data from urban Kenya. 2019;3.
14. Handebo SJOAJoC. Informed choice of contraceptive methods among women in Ethiopia: further analysis of the 2016 Ethiopian demographic health survey. 2021:83-91.
15. Ontiri S, Kabue M, Biesma R, Stekelenburg J, Gichangi PJPO. Assessing quality of family planning counseling and its determinants in Kenya: analysis of health facility exit interviews. 2021;16(9):e0256295.
16. Pradhan MR, Patel SK, Saraf AAJJoBS. Informed choice in modern contraceptive method use: pattern and predictors among young women in India. 2020;52(6):846-59.
17. Boadu IJRH. Coverage and determinants of modern contraceptive use in sub-Saharan Africa: further analysis of demographic and health surveys. 2022;19(1):1-11.
18. Blackstone SR, Nwaozuru U, Iwelunmor JJIqoche. Factors influencing contraceptive use in sub-Saharan Africa: a systematic review. 2017;37(2):79-91.
19. Zegeye AF, Negash WD, Kassie AT, Wassie LA, Tamir TTJPo. Home delivery among women who had optimal ANC follow-up in Sub-Saharan Africa: A multilevel analysis. 2023;18(11):e0295289.

20. Sommet N, Morselli D. Correction: Keep Calm and Learn Multilevel Logistic Modeling: A Simplified Three-Step Procedure Using Stata, R, Mplus, and SPSS. International Review of Social Psychology. 2017;30(1).
21. Belay DG, Aragaw FM, Teklu RE, Fetene SM, Negash WD, Asmamaw DB, et al. Determinants of inadequate minimum dietary diversity intake among children aged 6–23 months in sub-Saharan Africa: pooled prevalence and multilevel analysis of demographic and health survey in 33 sub-Saharan African Countries. Frontiers in Nutrition. 2022;9.
22. Tesema GA, Mekonnen TH, Teshale AB. Individual and community-level determinants, and spatial distribution of institutional delivery in Ethiopia, 2016: Spatial and multilevel analysis. PloS one. 2020;15(11):e0242242.
23. Dansereau E, Schaefer A, Hernández B, Nelson J, Palmisano E, Ríos-Zertuche D, et al. Perceptions of and barriers to family planning services in the poorest regions of Chiapas, Mexico: a qualitative study of men, women, and adolescents. 2017;14:1-10.
24. Hrusa G, Spigt M, Dejene T, Shiferaw SJPo. Quality of Family Planning Counseling in Ethiopia: Trends and determinants of information received by female modern contraceptive users, evidence from national survey data,(2014-2018). 2020;15(2):e0228714.
25. Loaiza E, Liang M, Snow R, editors. Informed choice for modern contraceptive use: evidence from 24 countries served by the UNFPA supplies programme. Population Association of America meeting; 2016.
26. Samuel B, Tsegaye B, Dulla D, Aynalem A, Israel E, Gebrie MJC, et al. Informed choice and its associated factors among women received immediate postpartum long-acting reversible contraceptives at public hospitals in Sidama Regional State, Ethiopia, 2022. 2023;8(1):32.
27. Hlongwa M, Tlou B, Hlongwana KJPAMJ. Healthcare providers' knowledge and perceptions regarding the use of modern contraceptives among adolescent girls in Umlazi Township, KwaZulu-Natal province, South Africa. 2021;38(1).
28. Apoorva Jadhav AJ, Vala-Haynes E. Informed choice and female sterilization in South Asia and Latin America. 2019.
29. Assaf S, Wang WenJuan WW, Mallick L. Provider counseling and knowledge transfer in health facilities of Haiti, Malawi, and Senegal. 2016.
30. Belachew TB, Negash WD, Bitew DA, Asmamaw DBJBPH. Prevalence of married women’s decision-making autonomy on contraceptive use and its associated factors in high fertility regions of Ethiopia: a multilevel analysis using EDHS 2016 data. 2023;23(1):83.
31. Yeboah A, Yaw D, Gyamfuah AJHJotOUoT. Influence of multiple media channels on adolescents’ knowledge and intentions to use contraceptives: a quantitative study of amasaman, the capital of ga west district assembly in Ghana. 2017;24(1):92-106.

Click here to access/download;Figure;INFORMED CHOICE 12.docx

Informed choice of modern Contraceptive Methods and determinant factors among reproductive age women in Eastern Africa countries: A Multilevel Analysis of Demographic and Health Survey.

Gebreeyesus Abera Zeleke[1*], Birtukan Atena Negash[1], Kassaw Belay Shiferaw[2], Berhan Tekeba[3], Tadesse Tarik Tamir[3], Workie Zemene Worku[4], Alebachew Ferede Zegeye[5].

[1] Department of Surgical Nursing, School of Nursing, College of Medicine and Health Science, University of Gondar, Gondar, Ethiopia.

[2] Department of physiotherapy, school of medicine College of Medicine and Health Sciences University of Gondar, Gondar, Ethiopia.

[3] Department of Pediatric and Child Health Nursing, School of Nursing, College of Medicine and Health Sciences, University of Gondar, Gondar Ethiopia.

[4] Department of Medical Nursing, School of Nursing, College of Medicine and Health Sciences University of Gondar, Gondar, Ethiopia.

[5] Department of community nursing, School of Nursing, College of Medicine and Health Science, University of Gondar, Ethiopia.

Correspondence Author*

Gebreeyesus Abera Zeleke

Department of Surgical Nursing, School of Nursing, College of Medicine and Health Science, University of Gondar, Gondar, Ethiopia.

Email address: gebreeyesusabe143@gmail.com

Co-authors

Birtukan Atena Negash

Email Address: birtukanatena@gmail.com

Kassaw Belay Shiferaw

Email address: uogbelay@gmail.com

Berhan Tekeba

Email address: berishboss7@gmail.com

Tadesse Tarik Tamir

Email address: Tadestar140@gmail.com

Workie Zemene Worku

Email Address: workiezemene@gmail.com

Alebachew Ferede Zegeye

Email address: alexferede24@gmail.com

Abstract

Background: Many contraceptive users discontinue their contraceptive methods or fail to use the service; consequently, unwanted pregnancy occurs and this has been public health problem in Sub-Saharan Africa countries especially eastern Africa countries. Informed choice of modern contraceptive method is an important indicator of family planning quality services. Evidence shows that informed choice of contraceptive methods lowers the potential risk of family planning discontinuation rate, misunderstanding of contraceptives method and unintended pregnancies finally lead to induced abortions. Therefore, the aim of this study was to ascertain the magnitude of informed choice of modern contraceptive methods user and its determinant factors among reproductive age women who are currently using selected modern in Easter African countries.

Methods: Data from Data from the recent DHS which was covered eight Eastern Africa countries from 2012 to 2020 was used for secondary data analysis. The total weighted sample was 6154 reproductive age women who used were modern contraceptive method. Stata version 14 was used to analysis secondary data. Determinants of informed choice were determined by using multilevel mixed-effects logistic regression model. Significant factors related with informed choice in multilevel mixed effect logistic regression model were decided when the p value of <0.05. The adjusted odds ratio (AOR) and confidence interval (CI) were used to interpret the outcome.

Result: In East Africa, the magnitude of informed choice found to be 20.70. determinant factors such maternal age (20 to 35) years (AOR=2.02 CI: 1.39, 2.93), (36-49) years (AOR =2.44, 95% CI: 1.66, 3.61), attending secondary & higher education (AOR =1.37 95% CI 1.02, 1.84), Media exposure (AOR = 1.25 95% CI 1.05, 1.49), visit health facility within 12 months were (AOR =1.31 95% CI: 1.11, 1.54) were significantly associated with an informed choice among reproductive age women modern contraceptive method user.

Conclusions and recommendation: This study finalized that an informed choice over eight Eastern Africa countries among reproductive age women who used modern contraceptive methods were low. Maternal age, attending secondary & higher education, Media exposure, visit health facility within 12 months were significantly associated with informed choice. Therefore, The Government, ministry of health and other stake holders in eastern Africa countries shall designing policies and strategies targeting informed choice modern contraceptive methods and above significant factors among reproductive age women from 15-49 years in Eastern Africa

Keywords: Informed choice, Contraceptive Methods, East Africa.

Background

Family planning access should be a fundamental component of services for sexual and reproductive health, according to in Cairo declaration, International Conference on population Development(1). Family planning comprise all of the services, policies, information, attitudes, practices, and commodities including contraceptives that enable individuals to avoid unintended pregnancy and make decisions and choose whether and/or when to have a child(2). Fundamentally, people have a human right to access contraceptive information and services, and high-quality contraceptive information and services support people's autonomy to choose the number and spacing of their children and provide a number of potential advantages, including improved maternal and child health(3). Informed choice is defined as the right of an individuals or couples to freely choose based on the options information well understanding possibly by comprehension knowledge(4). Many contraceptive users discontinue their contraceptive methods or fail to use consequently unwanted pregnancy occur and this was public health problem in Sub-Saharan Africa countries especially eastern Africa countries(5). Informed choice of modern contraceptive method is an important indicator of family planning quality services(6, 7). Evidence shows that informed choice of contraceptive methods lowers the potential risk of family planning discontinuation rate, dissatisfaction need of contraceptives methods, unintentional pregnancies finally lead to induced abortions(8, 9). Informed choice has played important role helping individuals to select a method that matches their needs and expectations to promote effective contraceptive use(10). Evidence on the prevalence of informed choice of modern contraceptive methods across different parts of the world revealed that very low. A study done in India 15.6%(11), Bangladesh 22%(12), Kenya 55.5%(13), Ethiopian (EDHS 2016) (36.2%)(14), in sub-Saharan Africa 49.47%(5). Contributing factors that studies previously on informed choice of contraceptive methods revealed that maternal age, Residence, types contraceptive use, wealth index, media exposure, maternal education, husband's occupation, source of contraceptive method, and visited health facility in the last 12 months were significantly contributed with informed choice of contraceptive methods(11, 14-16) Globally, different strategies have been applied to increase contraceptive utilization, but the prevalence of contraceptive methods in SSA remains unacceptably low and the misconception of contraceptive side effects is a major public health problem(17, 18). Even though studies have been conducted on the utilization, unmet need, and discontinuation of contraceptive methods in SSA countries, to our knowledge, evidence on informed choice of contraceptive methods in eastern

African region was very limited. Therefore, the aim of this study was to ascertain the magnitude of informed choice of modern contraceptive methods user and its determinants among women aged 15-49 years who are currently using selected modern such as (Pill, injectable, IUD, Norplant/ implant, female sterilization) in Easter African countries

Methods

Patient and public involvement statement

The data used in this study were secondary data from the Demographic and Health Surveys (DHS).

Study design, study area, and period

The recent demographic health survey Data from the eight East African countries which were conducted between 2012 and 2020 were used to carry out multilevel mixed effect analysis. To produce updated health and health-related indicators, the DHS is a community-based cross-sectional research data that is carried out every five years.

Data source, study population and sampling technique

The East African countries DHS datasets from 2012 to 2020 were used for the secondary data analysis. We used DHS surveys from Eight East African countries such as Burundi, Comoros, Ethiopia, Malawi, Rwanda, Uganda, Zambia, and Zimbabwe. The data were appended to filter out the magnitude and determinant factors of among reproductive age women who were informed choice among modern contraceptive usage in East African nations. The survey for every country contains different datasets. DHS deploys a stratified two-stage cluster design that includes enumeration areas as the first stage and generates a sample of households from each enumeration area as the second stage. The variable modern contraceptive (v312) from maternal recode (IR) data set was recoded to drop other than pill IUD, injectable, female sterilization, implant/norplant contraceptive methods. A binary logistic regression model was used to identify determinants a factor to informed choice of modern contraceptive users. Determinants of informed choice were reported in terms of an AOR with a significance level of (95%). In the univariate analysis, at 95% confidence intervals with a p-value of < 0.25 was considered a candidate for the multivariable analysis of data. All variables with p values <0.05 were considered statistically significant. A total weighted sample of 6154 women was included in the study (**Table 1**)

Study variable

Outcome variables: The outcome of this study was informed choice of modern contraceptive methods. Data on informed choices were collected among reproductive 15-49 years of age who used one of the six modern contraceptive methods (female sterilization, implant/Norplant, injectable contraceptive, IUD, pill and emergency contraceptive). It was constructed based on the responses provided to questions in the DHS questionnaire. Measurement of the outcome variables was determined by using computation four variables **(table 2).**

Independent Variable: Hence DHS data are hierarchical, independent factors come from two sources that was at individual and community levels were considered for this analysis. The individual-level independent variables were reproductive women's age (15-19, 20-35, 36-49), Maternal educational status (No formal education, Primary education , Secondary and Higher education), Maternal occupation (House wife, employee, Others,), Religion (Cristian, Muslim, Others), Wealth index (Poor, Middle, Distance to health facility (Big problem, Not a big problem), media exposure (yes, no), intermate utilization (yes, no), Residency (urban, rural), Husband educational status (No formal education, Primary education, secondary and higher education) Occupational status (No working, Employed, house wife, Other), Husband occupational status (No working, Employed, Other), Contraceptive Decision (women, husband/partner, Joint decision), Currently Contraceptive (No, Yes) Visiting health facility in 12 month (No, Yes)

The community-level variables: Community poverty level (Low, High), Community illiteracy level (Low, High), Community media exposure, (Low, High), Community distance from health facility (Low, High), Place of residency (Urban, rural) in East African countries.

Data processing and statically analysis

The data obtained from DHS data sets were cleaned, recorded, and analyzed using Stata version 14 statistical software. The DHS data's variables are organized in clusters, and those in a cluster are more similar to one another than those of other clusters. To run a standard logistic regression model, the assumptions of independent observations and equal variance across clusters were broken. This implies that using a sophisticated model to consider between-cluster factors is necessary. Given this, multilevel mixed-effects logistic regression was used to determine the factors that associated with modern contraceptive informed choice. Multilevel mixed effect logistic regression follows four models: the null model (only outcome variable), mode I (individual level variables), model II (community level variables), and model III (both individual and

community level variables). The null model without independent variables was used to check the variability of informed choice of modern contraceptive method across the cluster. The relationship of individual-level variables with outcome variable (Model I) and the association of community-level variables with outcome variable (Model II) were determined. In the final model, the association of both individual and community-level variables was fitted with the outcome variable.

Random effects

Random effects or measures of variation of the outcome variables were estimated by the median odds ratio (MOR), intra-class correlation coefficient (ICC), and proportional change in variance (PCV). The intra-cluster correlation coefficient (ICC) and proportional change in variance (PCV) were computed to measure the variation between clusters. Taking cluster as a random variable, the ICC reveals the variation of informed choice between clusters was calculated as; $ICC = \frac{VC}{VC+3.29} \times 100\%$. The MOR is the median value of the odds ratio between the area of the highest risk and the area of the lowest risk for informed choice of modern contraceptive when two clusters are randomly selected, using clusters as a random variable; MOR= $e^{0.95\sqrt{VC}}$. In addition to this, the PCV reveals the variation in the magnitude of informed choice of modern contraceptive usage explained by factors and computed as; $PCV = \frac{Vnull-VC}{Vnull} \times 00\%$; where Vnull = variance of the null model and VC = cluster level variance (19-21). The fixed effects were used to estimate the association between the likelihood of informed choice and individual and community level independent variables. It was assessed and presented using adjusted odds ratio (AOR) and 95% confidence intervals with a p-value of < 0.05. Because of the nested nature of the model, deviation = -2(log likelihood ratio) was used to compare models, and the model with the lowest deviance was selected as the best-fitted model. The variables used in the models were verified for multi-collinearity by measuring the variance inflation factors (VIF), with the findings falling within acceptable limits of 1-10.

Result

In this study, a total of 6154 reproductive age women who had informed choice of modern contraceptive user. More than half of women (68.73%) 20-35 years of old and more than two third (71.18%) was live in rural parties of East Africa and half of the participant was 51.9% had primary education and more than 90% of the participant were not used internet and more than half (65.45) of the women was visiting health facility within 12 months of the survey **(table 3)**

The magnitude of informed choice among reproductive age women who used modern contraceptive method such as pill, IUD, injectable, implant/Norplant in Eastern African countries found to be 20.70 (95% CI: (19.65, 21.78) **(figure 1)**

Random effect (Measures of variation) and model fitness

A null model was used to ascertain whether the data supported the decision to assess randomness of at the community level. Findings from the null model revealed that there were significant differences in informed choice between communities, with a variance of .0883961 and a P value of 0.000. The variance within clusters contributed 11.56% of the variation in cluster, while the variance between cluster 2.61%. In the null model, the odds of informed choice differed between higher and lower risk clusters by a factor of times. The intraclass correlation value for Model I indicated that 0.76% of the variation in informed choice accounts for the disparities between communities. Then, with the null model, we used community-level variables to generate Model II. Cluster variations were the basis for 16.61% of the differences in informed choice, according to the ICC value from Model II. In the final model (model III), which attributed approximately 79.44% of the variation in the likelihood of informed choice to both individual and community-level variables, the likelihood of informed choice varied by 1.69 times across low and high informed choice in clusters **(table 4)**

Determinants of individual and community level factors with informed choice of among reproductive age women who used modern Contraceptive in East Africa countries.

In the final fitted model of multivariable multilevel logistic regression, maternal age 20-35 years, 36-49 years, secondary and higher education, media exposure, visiting the health facility, were significantly associated with informed choice of modern contraceptive among reproductive age women in east African countries.

The odds of informed choice were 2.022 times higher among reproductive women aged 20 to 35 years compared to women aged 15to 19 years **(AOR** = 2.02 95% CI: 1.39-2.92). The odds of informed choice were 2.44 times more likely to occur among reproductive women age 15 to 19 years of age **(AOR**=2.44, 95% CI, 1.66-3.61) and the odds of informed choice were 1.37 times higher who had secondary and higher education level compared to women who had no any education **(AOR=**1.37 95% CI 1.02-1.84). A reproductive age woman who had media exposure were 1.25 times more likely to informed choice for modern contraceptive compared to a woman who had not media exposure **(AOR**=1.25 95% CI: (1.05-1.49). and women who were visiting health facility 1.32 times more likely informed choice for modern contraceptive compared to women who were no visiting health facility with in 12 months of study period **(AOR=**1.32 95% CI (1.11-1.54) **(table 6)**

Discussion

Informed choice of modern contraceptive method contributes significant problem among reproductive age women in developing countries mainly in Eastern Africa. The aim of this research was to ascertain the magnitude and determinant factors modern contraceptive informed choice among reproductive age women in Eastern Africa countries. In this study, the magnitude of informed choice among reproductive age women found to be 20.70 (95% CI: (19.65, 21.78). This finding revealed that substantial number of reproductive age women using modern contraception method were uninformed choice about contraceptive methods, side effects and other concerned issue of family planning, consequently, discontinuation of service use(22). This finding was lower than the previous studies conducted in sub-Saharan Africa 49.47%(5) , multilevel study in Ethiopia 36.2%(14), 30% (23) UNFPA study in 24 countries 52%(24) . This discrepancy might be due to the deference in socio-econommic and demographic condition, and variation in health infrastructure and health care service policy and the health service coverage, quality of maternal healthcare services, and economic and health policies of UNFPA the are better compared with those of Eastern Africa countries. On the other hand, this study was inline with study conducted southern Ethiopia , Sidama Region 23.5%(25)

In the final model of multilevel logistic regression analysis variables such as Maternal age, Maternal education, having media exposure, had visiting health facility within 12 months were found to be significantly associated with informed choice of modern contraceptive method user.

Given this, the odds of having an informed choice of modern contraceptive among women 20-35 and 36-49 years old were 2.02 times (AOR=2.02 CI: 1.39, 2.93) and 2.44 times (AOR =2.44, 95% CI: 1.66, 3.61) higher as compared to 15-19 years old women respectively. This find was consistent with study done in Ethiopia (14), sub-Saharan countries (5) , India(11). As the women's age increases, the chance of having an information, experience and learning about contraceptive also increases also one study conclude that, healthcare providers marginalize attitudes toward younger women(11, 26). At the same time, women who attend secondary & higher education were 1.37 times (AOR =1.37 95% CI 1.02, 1.84) higher odds of informed choice of modern contraceptive methods user compared to women who had no formal education. This is consistent, with studies done elsewhere (5, 11, 14, 27). The possible reason could be higher educated women more likely to engage with health care provider communication and understand information's they receive from their health care provider about informed choice of modern contraceptive, in addition, Educated women were better information access from other sources like media (15, 28). This result indicated that the importance of womens education for enhancing informed choice of modern contraceptive methods in East African countries.

Similarly, women who had media exposure were 1.25 times (AOR = 1.25 95% CI 1.05, 1.49) higher odds of informed choice of modern contraceptive methods user as compared to women who had no media exposure. This finding was similar with study done in Ethiopia (EDHS 2016) and sub Saharan Africa (5, 29). The possible justification for thus association was, women who had media exposure could be easily retained information about different types of contraceptive methods and their side effects, which enabled them to make an informed of contraceptive methods(30). In addition to this, women who visit health facility within 12 months were 1.31 times (AOR =1.31 95% CI: 1.11, 1.54) higher odds of informed choice of modern contraceptive methods user as compared to those who were not visiting health facility within 12 months of survey. This finding was consistent with study in Ethiopia (EDHS 2016). The justification of this finding could be, women's who had trained visiting health facility could easily obtain or access more information about different types of contraceptive methods, their preferable choice about contraception(30).

Strength and limitation

This study was national representative demographic and health survey data. Regarding limitations, as with any cross-sectional study, causal relationship was not determining and recall bias was not avoid, moreover, the finding on informed choice represents only selected modern contraceptive methods such as (pills, IUD, Implant/Norplant, injectable like Depo-Provera, female sterilization).

Conclusion and Recommendation

A substantial number of reproductive age women 15-49 years in East Africa were inadequately informed about modern contraceptive side effect and/or other available method. In this study only 20.7% of women were adequately informed choice about modern contraceptive side effect and available methods by healthcare providers. Maternal age, Maternal educational status, Media exposure, visiting health facility within 12 months were significantly associated with informed choice of contraceptive methods, Therefore, health-care facilities should focus on enabling women to make an informed choice of contraception method to secure the continual use of the service. Providers also shall use counseling sessions to help women make a thoughtful decision on contraceptives methods.

Declaration

Ethical approval and consent to participate

This study was based on analysis of existing survey datasets in the public domain (EDHS) that are freely available online with all the identifier information anonymized, no ethical approval was required.

Consent for publication

Not applicable

Data and material availability

Demographic and Health Survey were used, and it is publicly available online at (https://www.dhsprogram.com).

Competing interest

Authors Declared that, there is no competing interest.

Funding

Not applicable

Author's contributions

GAZ: involved in designing the study, data extraction, data analysis, interpretation, report and manuscript writing.

BAN: involved in data analysis, interpretation, and manuscript writing.

KBS: involved in data extraction, and manuscript writing.

BT: involved in analysis, and result interpretation.

TTT: involved in designing the study, interpretation, report and manuscript writing

WZW: involved in analysis, and result interpretation

AFZ: involved in designing the study, interpretation analysis and manuscript writing

Acknowledgements

We would like to say the DHS programs for allowing us to use the relevant DHS dataset in this study.

Reference

1. Rimon JG, Tsui AOJGHS, Practice. Regaining momentum in family planning. 2018;6(4):626-8.
2. Starbird E, Norton M, Marcus RJGhs, practice. Investing in family planning: key to achieving the sustainable development goals. 2016;4(2):191-210.
3. Ali M, Tran NTJBS, Health R. Defining counselling in contraceptive information and services: outcomes from an expert think tank. British Medical Journal Publishing Group; 2021.
4. Newman K, Feldman-Jacobs CJW, DC: Population Reference Bureau. Family Planning and Human Rights–What's the connection and why is it important. 2015.
5. Tsega NT, Haile TT, Asratie MH, Belay DG, Endalew M, Aragaw FM, et al. Pooled prevalence and determinants of informed choice of contraceptive methods among reproductive age women in Sub-Saharan Africa: A multilevel analysis. 2022;10:962675.
6. Hancock NL, Stuart GS, Tang JH, Chibwesha CJ, Stringer JS, Chi BHJC, et al. Renewing focus on family planning service quality globally. 2016;1:1-6.
7. Polis C, Bradley SE, Bankole A, Onda T, Croft TN, Singh S. Contraceptive failure rates in the developing world: an analysis of demographic and health survey data in 43 countries. 2016.
8. Chakraborty NM, Chang K, Bellows B, Grépin KA, Hameed W, Kalamar A, et al. Association between the quality of contraceptive counseling and method continuation: findings from a prospective cohort study in social franchise clinics in Pakistan and Uganda. 2019;7(1):87-102.
9. Kisuza RK, Kicaber S, Bary DA, Bongomin F, Garimoi COJm. Factors associated with contraceptive failure in Uganda: Analysis of the 2016 Uganda Demographic and Health Survey. 2022:2022.02. 09.22270716.
10. Dehlendorf C, Krajewski C, Borrero SJCo, gynecology. Contraceptive counseling: best practices to ensure quality communication and enable effective contraceptive use. 2014;57(4):659.
11. Jain AKJJobs. Information about methods received by contraceptive users in India. 2017;49(6):798-810.
12. Hossain S, Sripad P, Zieman B, Roy S, Kennedy S, Hossain I, et al. Measuring quality of care at the community level using the contraceptive method information index plus and client reported experience metrics in Bangladesh. 2021;11.
13. Feeser K, Chakraborty NM, Calhoun L, Speizer ISJGor. Measures of family planning service quality associated with contraceptive discontinuation: an analysis of Measurement, Learning & Evaluation (MLE) project data from urban Kenya. 2019;3.
14. Handebo SJOAJoC. Informed choice of contraceptive methods among women in Ethiopia: further analysis of the 2016 Ethiopian demographic health survey. 2021:83-91.
15. Ontiri S, Kabue M, Biesma R, Stekelenburg J, Gichangi PJPO. Assessing quality of family planning counseling and its determinants in Kenya: analysis of health facility exit interviews. 2021;16(9):e0256295.
16. Pradhan MR, Patel SK, Saraf AAJJoBS. Informed choice in modern contraceptive method use: pattern and predictors among young women in India. 2020;52(6):846-59.

17. Boadu IJRH. Coverage and determinants of modern contraceptive use in sub-Saharan Africa: further analysis of demographic and health surveys. 2022;19(1):1-11.
18. Blackstone SR, Nwaozuru U, Iwelunmor JJIqoche. Factors influencing contraceptive use in sub-Saharan Africa: a systematic review. 2017;37(2):79-91.
19. Sommet N, Morselli D. Correction: Keep Calm and Learn Multilevel Logistic Modeling: A Simplified Three-Step Procedure Using Stata, R, Mplus, and SPSS. International Review of Social Psychology. 2017;30(1).
20. Belay DG, Aragaw FM, Teklu RE, Fetene SM, Negash WD, Asmamaw DB, et al. Determinants of inadequate minimum dietary diversity intake among children aged 6–23 months in sub-Saharan Africa: pooled prevalence and multilevel analysis of demographic and health survey in 33 sub-Saharan African Countries. Frontiers in Nutrition. 2022;9.
21. Tesema GA, Mekonnen TH, Teshale AB. Individual and community-level determinants, and spatial distribution of institutional delivery in Ethiopia, 2016: Spatial and multilevel analysis. PloS one. 2020;15(11):e0242242.
22. Dansereau E, Schaefer A, Hernández B, Nelson J, Palmisano E, Ríos-Zertuche D, et al. Perceptions of and barriers to family planning services in the poorest regions of Chiapas, Mexico: a qualitative study of men, women, and adolescents. 2017;14:1-10.
23. Hrusa G, Spigt M, Dejene T, Shiferaw SJPo. Quality of Family Planning Counseling in Ethiopia: Trends and determinants of information received by female modern contraceptive users, evidence from national survey data,(2014-2018). 2020;15(2):e0228714.
24. Loaiza E, Liang M, Snow R, editors. Informed choice for modern contraceptive use: evidence from 24 countries served by the UNFPA supplies programme. Population Association of America meeting; 2016.
25. Samuel B, Tsegaye B, Dulla D, Aynalem A, Israel E, Gebrie MJC, et al. Informed choice and its associated factors among women received immediate postpartum long-acting reversible contraceptives at public hospitals in Sidama Regional State, Ethiopia, 2022. 2023;8(1):32.
26. Hlongwa M, Tlou B, Hlongwana KJPAMJ. Healthcare providers' knowledge and perceptions regarding the use of modern contraceptives among adolescent girls in Umlazi Township, KwaZulu-Natal province, South Africa. 2021;38(1).
27. Apoorva Jadhav AJ, Vala-Haynes E. Informed choice and female sterilization in South Asia and Latin America. 2019.
28. Assaf S, Wang WenJuan WW, Mallick L. Provider counseling and knowledge transfer in health facilities of Haiti, Malawi, and Senegal. 2016.
29. Belachew TB, Negash WD, Bitew DA, Asmamaw DBJBPH. Prevalence of married women's decision-making autonomy on contraceptive use and its associated factors in high fertility regions of Ethiopia: a multilevel analysis using EDHS 2016 data. 2023;23(1):83.
30. Yeboah A, Yaw D, Gyamfuah AJHJotOUoT. Influence of multiple media channels on adolescents' knowledge and intentions to use contraceptives: a quantitative study of amasaman, the capital of ga west district assembly in Ghana. 2017;24(1):92-106.
