# Supplementary material for: Informed choice of modern Contraceptive Methods and determinant factors among reproductive age women in Eastern Africa countries: A multilevel analysis of demographic and health survey
Source: PLoS One. 2025 May 19;20(5):e0323797. doi: 10.1371/journal.pone.0323797 (PMC12088018; doi:10.1371/journal.pone.0323797)
Supplement: Attachment — Submitted filename: Peer_Review contraception.docx [file pone.0323797.s003.docx]

**Date:** Thursady, December 6th, 2024

**Journal:** PLOS ONE

Dear Editor-in-Chief, Associate Editor, and Authors

Thank you for the opportunity to review the manuscript entitled "**Informed choice of modern Contraceptive Methods and determinant factors**

**among reproductive age women in Eastern Africa countries: A Multilevel**

**Analysis of Demographic and Health Survey.**".

- **Comments to Authors:**

**Abstract:**

- please correct the linguistic error of repetition in the following phrase “**Data from** Data from the recent DHS” stated in the subtitle “method”.

**Title:**

- The title of the study is clear, inclusive and precise to the study’s objectives and aims. The authors stated the study design distinctly. However, concerns are regarding using “modern” description for a well-known methods of contraception.

**Introduction:**

- The rationale and aim of the study are well stated.
- The introduction would benefit from a more comprehensive description of how family planning services are being delivered in the research area; this would help make the context clearer for readers and researchers unfamiliar with Sub-Saharan Africa.
- While the idea of informed choice when choosing contraception method has been stated several times as an important factor, further elaboration is needed on its broader effects, beyond just preventing unwanted pregnancies. Specifically, a more direct link between informed choice and lower contraceptive discontinuation rates would strengthen the argument.
- The inclusion of statistical data from countries like India, Bangladesh, Kenya, Ethiopia, and sub-Saharan Africa adds strength to the argument. However, there is a lack of explanation about these statistics. How were these numbers gathered? Clarifying how informed choice was ensured and its relationship to better contraceptive choices would add credibility and impact to the discussion.

**Methods:**

- The methods section provides a comprehensive description of the study design, data source, population, and statistical analysis. Moreover, Tables summarizing key variables are quiet helpful.
- The authors clearly explain the weighted sample size, which helps strengthen the reliability of the method.
- The explanation of the statistical models (e.g., multilevel mixed-effects logistic regression) is detailed but might be difficult to follow for readers unfamiliar with advanced statistical methods.
- Also, the use of specialized terms such as "proportional change in variance" (PCV), "intra-class correlation coefficient" (ICC), and "variance inflation factors" (VIF) may be challenging for some readers. It would be beneficial to define these terms more clearly, when they first appear or provide references for readers unfamiliar with them.
- Regarding “outcome” variables, the authors conducted a questionnaire survey that would define and assess “informed choice” such as; knowing the side effects and other available methods. Nevertheless, many other aspects are worth mentioning in order to label it as “informed choice”. These aspects includes but **not** limited to:
  - Access to information: providing clear details about each method, each method , its effectiveness, side effects, and any medical or personal considerations.
  - **Understanding Options**: Ensuring that individuals understand the information provided and can ask questions to clarify any uncertainties.
  - **Freedom of choice:** must have the autonomy to choose the method that best suits their personal needs and preferences without any pressure from healthcare provider or a family member.
- While the methodology is well-detailed, the section could benefit from a brief mention of potential limitations in the way informed choice is measured.
- Overall, the methods section is well-organized and provides a thorough description of the study's design and analysis. With a few clarifications and improvements, it would be more accessible to a wider audience.

**Results:**

- The authors conducted a thorough analysis of the results, supported by sufficient amount of data justifying the conclusion.
- The data are clearly presented by the authors, and the use of percentages for each variable enhances the clarity of the findings. The study's main result, showing the magnitude of informed choice among women using modern contraceptives, is well articulated (20.70%).

**Discussion:**

- The author effectively highlights key findings and discusses the demographic and socio-economic factors that contribute to this low rate.
- The comparison with other studies in sub-Saharan Africa, such as those in Ethiopia, India, and broader African contexts, is useful in contextualizing the results and offering possible explanations for the discrepancies observed.
- While the authors mentioned multiple contributing socio-economic factors, it would be beneficial to delve deeper into how specific health policies or community-level interventions could address the lack of informed choice, particularly in rural areas. This could provide actionable insights for policymakers.
- Overall, the discussion section is informative and offers a solid interpretation of the study results.

**Strength and Limitation:**

- The acknowledgment of limitations such as the potential for recall bias demonstrates transparency and a clear understanding of the study's constraints. This might provide a guidance for future researches.

**Conclusion:**

- Correctly answered the research question.
- The authors effectively highlights the factors associated with informed choice, such as maternal age, education, media exposure, and health facility visits.
- Including recommendations and suggestions for improving informed choice aimed at healthcare policymakers and media representatives would enhance the conclusion.

**Minor:**

- Some minor grammatical issues could be addressed to improve readability.
- English editing and proofreading by a native English speaker needed. The authors should correct some typos and language mistakes.
