# Supplementary material for: Informed choice of modern Contraceptive Methods and determinant factors among reproductive age women in Eastern Africa countries: A multilevel analysis of demographic and health survey
Source: PLoS One. 2025 May 19;20(5):e0323797. doi: 10.1371/journal.pone.0323797 (PMC12088018; doi:10.1371/journal.pone.0323797)
Supplement: Attachment — Submitted filename: Informed choice PBP complated NNN1.docx [file pone.0323797.s006.docx]

**Response to Editor and reviewers' comments**

**ID:** PONE-D-24-06637

**Title:** Informed choice of modern Contraceptive Methods and determinant factors among reproductive age women in Eastern Africa countries: A Multilevel Analysis of Demographic and Health Survey.

**Journal:** PLOS ONE

**Name of Authors: Gebreeyesus Abera Zeleke^1^*, Birtukan Atena Negash^1^, Kassaw Belay Shiferaw^2^, Berhan Tekeba^3^, Tadesse Tarik Tamir^3^, Workie Zemene Worku^4^, Alebachew Ferede Zegeye^5.^**

**Subject:** Submission of revised manuscript

Yours sincerely

**Gebreeyesus Abera Zeleke (on behalf of all authors) university of Gondar, Gondar Ethiopia**

**Response to Editors and reviewer comments**

We greatly appreciate the feedback that the editor and reviewers of this manuscript have given us. The comments were encouraging and inspiring and the review appears to share our judgment that this study and its results are clinically important. Please see below, our detailed response to the comments. Also, kindly find the attached file of the unmarked version of the revised manuscript and the revised manuscript with track changes that are uploaded separately and point by point response that the editor and reviewers’ questions, suggestion and recommendation are uploaded separately.

**Response to editor comments**

**INSTRUCTION**
#1. Please include the following items when submitting your revised manuscript:

- A rebuttal letter that responds to each point raised by the academic editor and reviewer(s). You should upload this letter as a separate file labeled 'Response to Reviewers'.
- A marked-up copy of your manuscript that highlights changes made to the original version. You should upload this as a separate file labeled 'Revised Manuscript with Track Changes'.
- An unmarked version of your revised paper without tracked changes. You should upload this as a separate file labeled 'Manuscript'.

**Response:** Dear editor, thank you for the recommendations. The corresponding author has uploaded a separate file; such as point by points response, a marked-up copy or Manuscript with Track Changes' and unmarked version of the revised manuscript. Kindly see uploaded files in system submission.

#2. Please ensure that your manuscript meets PLOS ONE's style requirements, including those for file naming.

**Response**: Dear editor, thank you for the recommendations. The authors ensured that the manuscript meeting PLOS ONE's style requirements. Kindly see the main body of manuscript such as abstract, the introduction, the method section, result and the discussion section.

#3. Please provide a complete Data Availability Statement in the submission form, ensuring you include all necessary access information or a reason for why you are unable to make your data freely accessible. If your research concerns only data provided within your submission, please write "All data are in the manuscript and/or supporting information files" as your Data Availability **Response**: Dear editor, thank you for the direction. The authors ensured that complete Data Availability Statement in the submission form; Demographic and Health Survey were used in this study, and it is publicly available online at (https://dhsprogram.comhttps://www.dhsprogram.com). Kindly see in the submission form and in manuscript in declaration section on page line number

#4.  PLOS requires an ORCID ID for the corresponding author in Editorial Manager on papers submitted after December 6th, 2016. Please ensure that you have an ORCID ID and that it is validated in Editorial Manager. To do this, go to ‘Update my Information’ (in the upper left-hand corner of the main menu), and click on the Fetch/Validate link next to the ORCID field. This will take you to the ORCID site and allow you to create a new ID or authenticate a pre-existing ID in Editorial Manager.

**Response**: Dear editor, thank you for the direction. The authors ensured that, the corresponding author ORCID ID <https://orcid.org/0009-0004-8978-8910>. please kindly see, authenticate a pre-existing ID in Editorial Manager.

#5. Please amend either the abstract on the online submission form (via Edit Submission) or the abstract in the manuscript so that they are identical.

**Response**: Dear editor, thank you for your direction. Accordingly, we correct our abstract the identical in submission main body of the abstract and on online submission form. Please kindly, see the amendment on abstract section both in revised manuscript and online submission form.

#6. Please include a separate caption for each figure in your manuscript.

**Response:** Dear editor, thank you for your direction. Accordingly, the authors ensured that Figures separate caption for each figure in our manuscript. Kindly see the amendment in result section on page 12-line number 236-238.

#7. We note you have included a table to which you do not refer in the text of your manuscript. Please ensure that you refer to Table 5 in your text; if accepted, production will need this reference to link the reader to the Table

**Response**: Dear editor, thank you for your detail editing our manuscript, we ensured that, table 5 was incorrectly cited. Now, we merge in to table 4. Please kindly see amendment on result section, on page 12-line number 252-255.

**Additional Editor Comments:**

#1. You reported an “informed choice” prevalence of 20.70% from DHS data spanning 2012 to 2020 across eight East African countries. Meanwhile, a study covering nine East African countries (DHS 2015–2021, DOI: 10.1371/journal.pone.0297018) reported an overall modern contraceptive prevalence of 45.68%. How do you explain such a significant gap between informed choice and modern contraceptive use, given that family planning counselling is expected to ensure informed decision-making at initiation?

**Response:** Dear editor, thank you for your critical and insightful comment, the significant gap between the prevalence of informed choice (20.70%) and modern contraceptive use (45.68%) may be explained by variations in the differences of these indicators and the impact of underlying contextual factors. Informed choice, as measured in our study, requires that women be counseled on five key aspects such as alternative methods, potential side effects, and how to manage side effects and others condition of selected modern contraceptive methods. This basic criterion reflects the quality of family planning counseling, which may not always be consistently implemented across health systems in the region. In contrast, modern contraceptive prevalence represents the proportion of women using any modern contraceptive method, regardless of whether their decision was fully informed. Several factors contribute to this discrepancy: limited trained healthcare workers, high client-to-provider ratios, and other barriers such as time constraints for counseling, which can impede comprehensive information. Additionally, education may affect women's ability to seek or understand family planning information, leading to modern contraceptive use without meeting the standards of informed choice.

#2. Your study excluded Madagascar and Tanzania but included Comoros. What was the rationale behind selecting these countries? Was it related to data availability, methodological concerns, or another reason?

**Response**: Dear editor, thank you for your detailed and valuable comment, as per your comment, the rationale behind selecting eight countries was due to data availability. Madagascar and Tanzania were excluded in our study due to data incompleteness. In the process of determining our outcome variable we compile the five variables such as v3a02, v3a03, v3a04 v3a05 v3a06. However, Tanzania, Kenya and Madagascar national DHS (v3a04 and v3a06) were incomplete data.

#3. You used DHS data from 2012 to 2020 despite the availability of more recent data (2015–2021). Why did you choose an earlier timeframe instead of leveraging the latest data?

**Response**: Dear editor, thank you for your valuable comment and for raising pertinent point regarding the data timeframe. The main reason, we chose 2012–2020 DHS dataset timeframe was **Completeness and Consistency of Data.** The 2012–2020 period provided a more complete and consistent data set across the countries included in our analysis. Some countries either did not have data available for the 2015–2021 period or had incomplete surveys, making the 2012–2020 period the most reliable and comprehensive option for our study. According to DHS guide statistics version 2, some countries DHS data were inconsistent and incomplete dataset. For instance, Tanzania and Kenya etc.

#6. Could this choice affect the relevance of your findings? Please elaborate further on how your study adds new insights to the existing body of knowledge on family planning and informed choice. What gaps does it fill over the existing data?

**Response**: Dear editor, thank you for your critical question, this study adds new insights to the existing body of knowledge on family planning and informed choice by addressing gaps in context-specific data, the importance of education and information availability, and the impact of gender roles in decision-making, this study contributes fresh perspectives to the body of knowledge already available on family planning and informed choice. It looks at how several elements, like psychological aspects and long-term changes in circumstances, affect people's capacity to make well-informed decisions regarding family planning. By combining these components, the study fills in the gaps left by earlier research that frequently ignores particular cultural settings and the caliber of the information offered, providing a better understanding of the obstacles and enablers to informed choice.

#7. Could you explain more clearly how you defined and operationalized your outcome variables? This clarification will help in understanding the study's methodology and its implications.

**Response**: Dear editor, Thank you for your comment. According to our research, "informed choice" refers to the process by which a person chooses modern contraceptive method of after being provided with pertinent, accurate, and easily understandable information about family planning methods such as female sterilization, pill, IUD, injectables, and implants. This information involves; all available method, the potential side effects and what they should do if they encounter side effect and informed by health care provider etc. [**Guide to DHS Statistics DHS-7 (version 2].** Kindly see amendment on method section on page 7-8-line number 172-173.

**Reviewers' comments:
Reviewer's Responses to Questions**

Reviewer #1: Dear author
Conducting a study on contraceptive methods is required in all countries. Therefore, conducting this study is commendable. Below are comments to improve the article.

**Abstract:**
#1. Please correct this sentence:
This study concluded that the proportion of reproductive age women using modern contraceptive techniques made an informed choice in eight nations in Eastern Africa.

**Response:** Dear reviewer, First and foremost, thank you for your insightful comments. We have corrected the above sentence. Kindly see the amendments in the revised manuscript on abstract section on page number 2, line number 76-77.

**Introduction**
#2. Do you have statistics on unwanted pregnancies in East Africa? If you have statistics, please write.

**Response**: Dear reviewer thank you for your comment, as per your recommendation, we incorporate information about unwanted pregnancies and illegal abortions in introduction section. kindly see the amendment on introduction section on page 3-line number 89-95

#3. It seems that illegal abortions following uninformed choice of contraceptive methods are your concern and this is the necessity of your study. Please explain more about this issue. Explain that illegal abortions threaten women's reproductive health.

**Response**: Dear reviewer thank you for your comment, our concern was how many reproductive age women were properly Informed choice at the time of starting modern contraceptive; about contraceptive side effect and other alternative methods. Providing autonomy for the family planning users.

**Methods**:

4. Barrier methods and vasectomy methods are not among the methods?

**Response**: Dear reviewer thank you for your constructive comment, in DHS the survey data includes Only users of female sterilization, pill, IUD, injectables and implants; in fact, IUD used as a Barrie methods and vasectomy methods are has not yet include because it is IR DHS data **(Guide to DHS Statistics DHS-7 (version 2).**

**Discussion**
5. Why is there no discussion of non-significant variables?

**Response**: Dear reviewer, thank you for your broad and detail review of our manuscript. Because; non-significant variables were not kay factors of our study, to manage our resource and to achieve our objective, we have to focus on only significant variables. In addition; non-significant variables do not give strong evidence to support meaningful conclusion about their impact on outcome variable.

**Reviewer #2: Comments to Authors:**

Abstract:

#1. Please correct the linguistic error of repetition in the following phrase “Data from Data from the recent DHS” stated in the subtitle “method”

**Response**: Dear reviewer thank you for your comment, as per your direction, we have corrected the above linguistic error; pleas kindly see the amendment on revised manuscripts under abstract section on method part on page number 2-line number 64-65.

#2. Title: The title of the study is clear, inclusive and precise to the study’s objectives and aims.

**Response**: Dear reviewer thank you for your constructive comment; in fact, your comment makes me inspiring and encouraging.

#3. The authors stated the study design distinctly. However, concerns are regarding using “modern” description for a well-known method of contraception.

**Response**: Dear reviewer thank you for your valuable comment; according Demographic and Health Surveys Data, family planning methods may be classified into modern and traditional. Traditional methods include withdrawal, breast–feeding and rhythm method. Whereas modern methods include condoms, hormonal contraceptives, Intra uterine device (IUD), implants and female sterilization. (**Guide to DHS Statistics DHS-7 (version 2)**

**Introduction:**

#4. The rationale and aim of the study are well stated.

**Response**: Dear reviewer thank you for your valuable comment.

#5. The introduction would benefit from a more comprehensive description of how family planning services are being delivered in the research area; this would help make the context clearer for readers and researchers unfamiliar with Sub-Saharan Africa. While the idea of informed choice when choosing contraception, method has been stated several times as an important factor, further elaboration is needed on its broader effects, beyond just preventing unwanted pregnancies. Specifically, a more direct link between informed choice and lower contraceptive discontinuation rates would strengthen the argument.

**Response**: Dear reviewer, thank you for your fundamental comment, as per your guide we amend the introduction. Please kindly see the amendment on introduction section on page number 3-line number 90-96.

#6. The inclusion of statistical data from countries like India, Bangladesh, Kenya, Ethiopia, and sub-Saharan Africa adds strength to the argument. However, there is a lack of explanation about these statistics. How were these numbers gathered? Clarifying how informed choice was ensured and its relationship to better contraceptive choices would add credibility and impact to the discussion.

**Response:** Dear reviewer, thank you for your insightful feedback. The Demographic and Health Surveys (DHS) in Bangladesh, Kenya, Ethiopia, and sub-Saharan Africa provided the statistical data cited. To ensure the inclusion of varied demographic groups, for example, data on the usage of contraceptives in Ethiopia were collected using nationally representative surveys with participants. Kindly see the amendment on page 5-line number 120-123.

**Methods:**
#7. The methods section provides a comprehensive description of the study design, data source, population, and statistical analysis. Moreover, Tables summarizing key variables are quite helpful. The authors clearly explain the weighted sample size, which helps strengthen the reliability of the method.

**Response**: Dear reviewer thank you for your valuable comment.

#8. The explanation of the statistical models (e.g., multilevel mixed-effects logistic regression) is detailed but might be difficult to follow for readers unfamiliar with advanced statistical methods.

Also, the use of specialized terms such as "proportional change in variance" (PCV), "intra-class correlation coefficient" (ICC), and "variance inflation factors" (VIF) may be challenging for some readers. It would be beneficial to define these terms more clearly, when they first appear or provide references for readers unfamiliar with them.

**Response**: Dear reviewer thank you for your critical concern. As per your comment, we have ensured to define the above specialized terms. Please kindly refer an explanation the unfamiliar terms on method section, on page number 8-9-line number 207-225.

#9. Regarding “outcome” variables, the authors conducted a questionnaire survey that would define and assess “informed choice” such as; knowing the side effects and other available methods. Nevertheless, many other aspects are worth mentioning in order to label it as “informed choice”. These aspects include but not limited to: Access to information: providing clear details about each method, each method, its effectiveness, side effects, and any medical or personal considerations. Understanding Options: Ensuring that individuals understand the information provided and can ask questions to clarify any uncertainties. Freedom of choice: must have the autonomy to choose the method that best suits their personal needs and preferences without any pressure from healthcare provider or a family member.

**Response**: Dear reviewer thank you for your critical concern. As per your comment, we have operationalized our out-outcome variable depending on **Guide to DHS Statistics DHS-7(version 2**; in fact, there are many scholars use the above terms as outcome variables other than DHS data, such as primary and other secondary data but many study’s use DHS were used the term informed choice.
10. While the methodology is well-detailed, the section could benefit from a brief mention of potential limitations in the way informed choice is measured.

**Response**: Dear reviewer thank you for your basic concern. As per your comment, we ensured that, the measurement of outcome variable was determined. Please kindly see the amendment on method section on page number 7-line number 172 on table 2.
11. Overall, the methods section is well-organized and provides a thorough description of the study's design and analysis. With a few clarifications and improvements, it would be more accessible to a wider audience.
**Results:**
• The authors conducted a thorough analysis of the results, supported by sufficient amount of data justifying the conclusion.
• The data are clearly presented by the authors, and the use of percentages for each variable enhances the clarity of the findings. The study's main result, showing the magnitude of informed choice among women using modern contraceptives, is well articulated 20.70%.

**Response**: Dear reviewer, thank you for your motivated comment.

**Discussion:**
 The author effectively highlights key findings and discusses the demographic and socio-economic factors that contribute to this low rate.

**Response**: Dear reviewer, thank you for your inspired comment
• The comparison with other studies in sub-Saharan Africa, such as those in Ethiopia, India, and broader African contexts, is useful in contextualizing the results and offering possible explanations for the discrepancies observed.
• While the authors mentioned multiple contributing socio-economic factors, it would be beneficial to delve deeper into how specific health policies or community-level interventions could address the lack of informed choice, particularly in rural areas. This could provide actionable insights for policymakers.
• Overall, the discussion section is informative and offers a solid interpretation of the study results.

**Response**: Dear reviewer, thank you for your comment
**Strength and Limitation:**
• The acknowledgment of limitations such as the potential for recall bias demonstrates transparency and a clear understanding of the study's constraints. This might provide a guidance for future researches.

**Response**: Dear reviewer, thank you for your feedback
**Conclusion:**
•Correctly answered the research question.
• The authors effectively highlights the factors associated with informed choice, such as maternal age, education, media exposure, and health facility visits.
• Including recommendations and suggestions for improving informed choice aimed at healthcare policymakers and media representatives would enhance the conclusion.

**Response**: Dear reviewer, thank you for your constructive comment
Minor:
• Some minor grammatical issues could be addressed to improve readability.
• English editing and proofreading by a native English speaker needed. The authors should correct some typos and language mistakes.

**Response**: Dear reviewer, thank you for your comment, as per your recommendation, we ensured that language mistakes, grammatical issues, typing error can corrected. Kindly see, revised manuscript and tracked change manuscript.

**Response to academic editors’ comments**

**A contraceptive method**
A contraceptive method is a technique or device used to prevent pregnancy. These methods include hormonal options like birth control pills, barrier methods like condoms, long-term options like IUDs, permanent solutions like sterilization, and natural methods such as fertility awareness."

**Dissatisfaction with contraceptive methods**

**Response:** Dear Academic Editor, thank you for your insightful comment**,** Dissatisfaction with contraceptive methods can arise due to various factors, including side effects, effectiveness, convenience, and personal preferences.

Independent factors come from two sources that was at individual and community levels were considered for this analysis

**Response:** Dear Academic Editor, thank you for your thoughtful comment. In multilevel analysis, community-level variables represent a characteristic or attributes of a group or cluster like community, or region that are used to explained variation in outcome variable. While individual level variable represents characteristic individual with in the particular group. To include community level variable, we must aggregate individual level data to the community level then treat these aggregated variables as a predictor in the multilevel model.

**Informed choice for modern contraceptive**

**Response:** Dear Academic Editor, thank you for your fundamental issue; Informed choice for modern contraceptives refers to an individual’s ability to select a contraceptive method based on complete and unbiased information, free from coercion. In our study, we assess informed choice by evaluating participants' knowledge of modern contraceptive methods, their understanding of benefits and side effects, and their autonomy in decision-making. This ensures that contraceptive use is based on personal preference rather than external pressure or misinformation. And proposition “ for” changes to of.

**A contraceptive method**

**Response:** Dear Academic Editor, thank you for your basic concern. Based on the comment. We try clarify contraceptive method refers to any approach used to prevent pregnancy, including modern methods and traditional methods. Modern contraceptive method includes hormonal contraceptives, IUDs, barrier methods, and sterilization and traditional methods includes withdrawal and fertility awareness. In our study, we focus on informed choice of modern contraceptive methods.

**Conclusion and Recommendation**

**Repose:** Dear Academic Editor, thank you for your comment, your comment guides to adjust Conclusion and Recommendation. kindly see the amendment on conclusion and recommendation section, on age 17-line number 332-342

**Patient and Public Involvement statement**

**Response:** Dear Academic Editor, thank you for your comment. In this study, the data were taken from Demographic and Health Surveys (DHS) data, which is secondary data. Pleas kindly see the amendment on page 6line number 139-141.

**Materials and methods**

**Response:** Dear Academic Editor, thank you for your constructive comment, we directly materials and method to “**Method”.** Kindly see amendment on method section on page number 6-line number 39.

**About ORCID ID**

**Response**: Dear editor, thank you for your comment to my ORCID ID, as per your direction, my ORCID ID information has been updated.

**Ethical statement issue**

**Response:** Dear editor, thank you for your comment, as per your guide, we review our ethical statement issue, however, ethical statement issue was in only in the method section. Kindly see the method section on page number 6-line number 139-225.

We are unable to open your Supporting Information file [Data usedfor analysis.rar]. Please kindly revise as necessary and re-upload.

**Response:** Dear reviewer, thank for your comment, we previously upload a zip file, unfortunately, the file has not open on your computer, there for it better to try upload in the form of excel files without change in to zipped file. Thank you for your understanding.
